# Electromagnetic radiation at 900 MHz induces sperm apoptosis through bcl-2, bax and caspase-3 signaling pathways in rats

**DOI:** 10.1186/s12978-015-0062-3

**Published:** 2015-08-04

**Authors:** Qi Liu, Tianlei Si, Xiaoyun Xu, Fuqiang Liang, Lufeng Wang, Siyi Pan

**Affiliations:** Key Laboratory of Environment Correlative Dietology, Ministry of Education, Wuhan, Hubei Province China; College of Food Science and Technology, Hua Zhong Agricultural University, Wuhan, Hubei Province China; Department of Bioengineering, Zhixing College of Hubei University, Wuhan, Hubei Province China

**Keywords:** Radiofrequency, Electromagnetic radiation, Sperm, Apoptosis, 900 MHz, Sprague-Dawley rats

## Abstract

**Background:**

The decreased reproductive capacity of men is an important factor contributing to infertility. Accumulating evidence has shown that Electromagnetic radiation potentially has negative effects on human health. However, whether radio frequency electromagnetic radiation (RF-EMR) affects the human reproductive system still requires further investigation. Therefore, The present study investigates whether RF-EMR at a frequency of 900 MHz can trigger sperm cell apoptosis and affect semen morphology, concentration, and microstructure.

**Methods:**

Twenty four rats were exposed to 900 MHz electromagnetic radiation with a special absorption rate of 0.66 ± 0.01 W/kg for 2 h/d. After 50d, the sperm count, morphology, apoptosis, reactive oxygen species (ROS), and total antioxidant capacity (TAC), representing the sum of enzymatic and nonenzymatic antioxidants, were investigated. Western blotting and reverse transcriptase PCR were used to determine the expression levels of apoptosis-related proteins and genes, including *bcl-2*, *bax*, *cytochrome c*, and *capase-3*.

**Results:**

In the present study, the percentage of apoptotic sperm cells in the exposure group was significantly increased by 91.42 % compared with the control group. Moreover, the ROS concentration in exposure group was increased by 46.21 %, while the TAC was decreased by 28.01 %. Radiation also dramatically decreased the protein and mRNA expression of bcl-2 and increased that of bax, cytochrome c, and capase-3.

**Conclusion:**

RF-EMR increases the ROS level and decreases TAC in rat sperm. Excessive oxidative stress alters the expression levels of apoptosis-related genes and triggers sperm apoptosis through bcl-2, bax, cytochrome c and caspase-3 signaling pathways.

## Background

Electromagnetic radiation (EMR) is emitted at a low-level radiofrequency (RF) between 800 and 2500 MHz. Accumulating evidence has shown that RF-EMR potentially produces negative effects on human health [1]. These effects are collectively called “microwave syndrome,” which manifest as headaches [2], miscarriage [3], elevated blood pressure [4], and even glioma [5]. Previous investigations revealed that RF-EMR with the same rate of energy deposited on the organism generate more biological effects compared with continuous microwave fields [6]. However, whether EMR affects human health remains controversial. Taking magnetic resonance imaging (MRI) and nuclear magnetic resonance (NMR) as examples, many investigations demonstrated that there were not any clinically relevant adverse effects on human health [7]. Nevertheless, some studies showed that NMR or MRI, a static and a gradient magnetic field combined with a radiofrequency (RF), would result in vertigo, nausea, reversible arrhythmia [8], and even deformity [9–11] or tumor [12, 13]. Thus, researchers have speculated that RF-EMR exerts harmful effects on living organisms and that organisms are highly sensitive to RF-EMR signals [14].

Despite the continued advances in science, the infertility rate is currently increasing because of increased stress factors. Approximately 14 % of couples in developed countries experience conception difficulties [15]. The decreased reproductive capacity of men is an important factor contributing to infertility, and RF-EMR may contribute to this condition [16]. However, whether RF-EMR affects the human reproductive system remains controversial [17]. Seze et al. found that exposure to EMR at a frequency of 900 MHz for 2 h/d exerted no significant effects on the concentrations of serum luteinizing hormone and follicle stimulating hormone [18]. Agarwal et al. reported no significant difference on total antioxidant capacity (TAC) and damage of DNA in ejaculated semen between the pilots who often use mobile phones and the control group [19]. Nevertheless, more studies have indicated that radiation intensity and duration of RF-EMR will influence the parameters of male reproduction capacity; these parameters include the concentration [20], mobility, morphology [21], viability of sperm cells [16, 20, 22] and sperm apoptosis [23]. Moreover, RF-EMR also affects or destroys leydig cells, which are adjacent to the seminiferous tubules [24], and decreases the diameter of seminiferous tubule [25] and the weight of testicular organs [26].

Two events can lead to reduction in reproductive capacity. One is sperm damage resulting from the thermal effect generated by the energy transfer of the RF electric field [27]. The other is DNA and protein damage resulting from elevated reactive oxidative species (ROS) level induced by RF-EMR [22, 28]. ROS can destroy unsaturated fatty acids and decrease the activity of enzymes related to antioxidation, such as superoxide dismutase (SOD) and glutathione peroxidase (GPX) [29].

Therefore, the present study investigated whether 900 MHz RF-EMR can trigger sperm cell apoptosis and affect semen morphology, concentration, and microstructure.

## Materials and methods

### Materials and chemicals

The Annexin V-FITC Apoptosis Detection kit was purchased from KeyGEN bioTECH Company (Nan Jing, Jiang Su, China). Human Tubal Fluid (HTF) was obtained from Millipore Co., Ltd. (Bedford, MA, USA). ROS, TAC (ABTS method), and bicinchoninic acid (BCA) kits were provided by Nanjing Jiancheng Bioengineering Institute (Nan Jing, Jiang Su, China). Polyclonal anti-cytochrome C, anti-caspase-3, anti-bcl-2, and anti-bax antibodies were purchased from Cell Signaling Technology Inc. (Danvers, Massachusetts, USA). Goat anti-rabbit IgG horseradish peroxidase (HRP) conjugate and enhanced chemiluminescence (ECL) kit were purchased from Wuhan Boster Biological Technology, Ltd. (Wuhan, China). PVDF membranes were purchased from Merck Millipore Inc. (Danvers, Massachusetts, USA). The mitochondrial protein extract kit was purchased from Beyotime Ltd. (Haimen, China). Trizol (Invitrogen, USA) was used for RNA extraction. The Ex TaqTM PCR kit was purchased from TAKARA Biotechnology Co., Ltd. (Dalian, China). SYBR Green/Flourescein qPCR Master Mix(2X) was purchased from Thermo Scientific Fermantas (USA).

### Animals

Ten-week-old male Sprague-Dawley rats weighing approximately 200 g obtained from Hubei Provincial Center for Disease Control and Prevention were stored in a room with a 12-h light:12-h dark photoperiod at a temperature of 20–22 °C, and a relative humidity of 50–60 %. Forty eight rats were randomly divided into the control and exposure groups of 24 each. The rats were fed in a single room and given free access to standard food and tap water, and then kept for 50 d to guarantee completion of the spermatogenic cycle (~42 d). At the end of treatment, all animals were euthanized by decapitation in accordance with the guidelines of The Institutional Animal Care and Use Committee of China. The trial and all research related to the trial was approved by the Academic Committee of Hua Zhong Agriculture University, Wuhan, Hubei province, China.

### Exposure system of electromagnetic field at 900 MHz

**Fig. 1:**
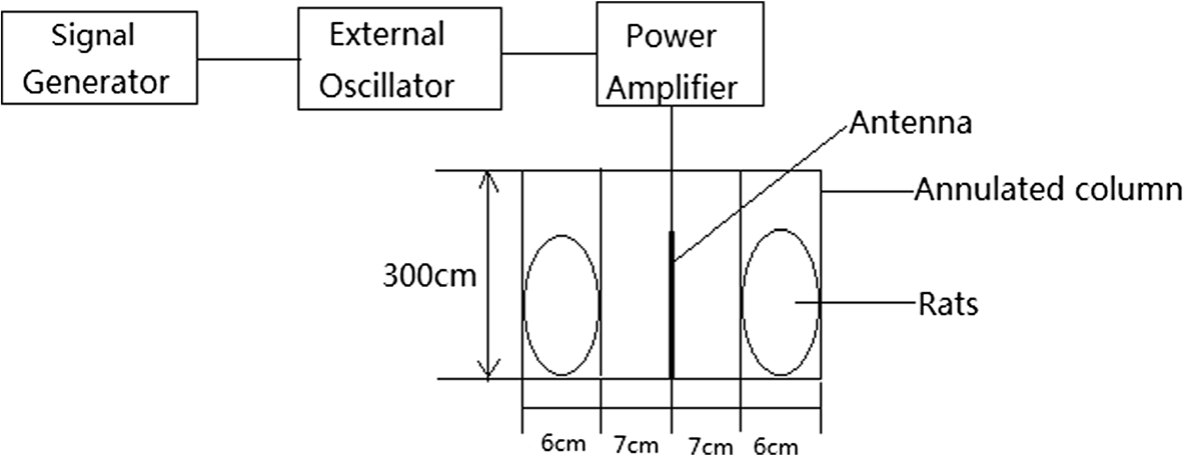
The sketch diagram of exposure setup

The exposure system of electromagnetic field was constituted with a signal generator, external oscillator, power amplifier and antenna. An electromagnetic wave of 900 MHz was generated by a signal generator (VP 8300, Panasonic, Japan). The amplitude modulation of sinusoidal waveform was obtained by an external oscillator (Philips-PM5127), amplified by a power amplifier (model AS0204-17R; Milmega Ltd., UK), and emitted by an antenna. The 900 MHz electromagnetic wave was modulated in amplitude with a frequency of 50Hz and 100 % modulation index by the exposure system mentioned above. The power density was measured by an RF-EMR strength meter (model TES92; TES Electrical Electronic Corp., Taiwan). The exposure setup is depicted in Fig. 1.

The holding units for the rats were customized in an annulated column with an inner diameter of 14 cm, an external diameter of 26 cm, and a height of 300 cm. The holding units were made of Plexiglas with a hole for air circulation. The antenna emitted electromagnetic wave in the center of the annulated column. The distance from the antenna to the samples was 7 cm to ensure the distribution of emitted power fell equally on all the animals.

The annulated column was divided into six equal parts. The rats in the exposure group were placed randomly in the six parts and exposed for 50 d at 2 h/d. The rats in the control group were kept in the same holding units for the same condition as the exposure group without electromagnetic radiation. The average power density of the equal parts was 1 ± 0.03 mW/cm^2^. The temperature of each part was tested during the exposure period. The RF-EMR produced at maximum an increase of temperature of about 0.07 °C, which indicated that the intensity of EMR in this study was too low to induce a thermal change. The whole-body specific absorption rate (SAR) was estimated in a prolate spheroid model with semimajor axis (a) and semiminor (b) [30]. The SAR value was calculated by the equations below when the rats were exposed on the RF-EMR with a power density of 1 mW/cm^2^:

1 $$ \mathrm{b}=\sqrt{\frac{3m}{4\rho \pi a}} $$

2 $$ {\mathrm{A}}_1=-0.994-10.690a+0.172a/b+0.739{a}^{-1}+5.660a/{b}^2 $$

3 $$ {A}_2=-0.914+41.100a+399.170a/b-1.190{a}^{-1}-2.141a/{b}^2 $$

4 $$ {A}_3=4.822a-0.084a/b-8.733{a}^2+0.002{\left(a/b\right)}^2+5.369{a}^3 $$

5 $$ {f}_0(MHz)=275\times {\left[8{a}^2+\pi \left({a}^2+{b}^2\right)\right]}^{-1/2} $$

6 $$ \mathrm{S}\mathrm{A}\mathrm{R}\left(\mathrm{W}/\mathrm{kg}\right) = \frac{A_1{\left(f/{f}_0\right)}^2\left(1+{A}_3\times f/{f}_0\right)}{10^3{\left(f/{f}_0\right)}^2+{A}_2\left({f}^2/{f_0}^2-1\right)} $$

In Eq. 1, the density of the rats (*ρ*) were assumed as 1 g/cm^3^. The value of *f* in Eq. 6 was 900 MHz. The length and weight of rats were tested and the SAR value was calculated by the equations above. The average SAR value was 0.66 ± 0.01 W/kg.

### Sperm sample preparation

The body and epididymal weights of the rats were detected after 50 d. The sperm sample from the epididymis was diluted to 10^7^/mL with HTF medium and held at 37 °C. The sperm samples from one epididymis of the rats were used for sperm counting, light microscopy, transmission electron microscopy (TEM), ROS testing, and TAC testing. The sperm samples from the other epididymis of the rats were used for western blot and RT-PCR analyses.

### Sperm count and microscopic observation of sperm morphology

Sperm samples were counted under the optical microscope (Nikon, Japan) by three people using a cytometer with a capillary to ensure the accuracy of the experiments. The results were obtained as sperm cell/mL.

A drop of each prepared sperm sample was uniformly placed on a slide and observed by three people under a light microscope. A total of 200 sperm cells per slide in 20 views were evaluated with a Nikon microscope (Nikon, Japan). The percentages of sperm cells with malformed head (headless, broken head, small head, large heads, no hook-shape) and tail (bent tails, coiled bodies, tubbiness) were recorded [20].

### TEM

The semen sample was fixed overnight at 4 °C with sodium cacodylate buffer containing 2.5 % glutaraldehyde. The sample was washed three times with 0.1 M phosphate-buffered saline (PBS), fixed with osmium tetroxide for 1 h, dehydrated with different ethanol concentrations, embedded in resin, and then sectioned using an ultramicrotome. The ultrathin sections were stained with uranyl acetate and lead citrate, and observed with H-7650 (Hitachi, Japan).

### Sperm cell apoptosis

The prepared semen samples were digested by 0.25 % pancreatic enzymes without ethylene diamine tetraacetic acid and then centrifuged at 1500 rpm for 5 min. The deposits were washed twice with PBS and resuspended in 500 μL of binding buffer. The suspension was incubated with 5 μL of Annexin V-FITC and 5 μL of propidium iodide at room temperature for 5–15 min and then directly detected by FACS Calibur flow cytometry (Becton-Dickinson, Franklin, NJ).

### Measurement of ROS and TAC in sperm samples

Sperm samples (1 mL) were centrifuged at 1000 g for 5 min. The precipitate (sperm) was washed twice with PBS and incubated with 10 μM DCFH-DA at 37 °C for 1 h. The sperm cells were washed twice with PBS and collected by centrifugation at 1000 g for 5 min. The precipitate was suspended with PBS and detected on an F-4600 spectrofluorophotometer (Hitachi, Japan) at an excitation wavelength of 500 nm and an emission wavelength of 530 nm. The TAC value in the sperm samples was measured at 540 nm on a UV-Vis spectrophotometer (721, Shanghai Optical Instrumental Factory, Shanghai, China) using the TAC assay kit in accordance with the manufacturer’s protocol. The results are shown as IU/mg protein. The protein contents of sperm samples were detected by the BCA protein assay kit (Nanjing Jiancheng, China) in accordance with the manufacturer’s protocol.

### Western blot of apoptosis-related proteins

After centrifugation at 500 g for 5 min, sperm cells were washed twice with PBS. Total protein was extracted using RIPA lysis buffer, and mitochondrial protein was extracted using the mitochondrial protein extract kit (Beyotime, China). Protein concentration was determined with the BCA kit in accordance with the manufacturer’s instructions.

For western blot analysis, 50 μg of solubilized sperm protein was loaded in a 12 % SDS-PAGE gel for electrophoresis. Cytochrome c proteins were transferred onto the PVDF membrane at 200 mA for 30 min; the bax and bcl-2 proteins at 200 mA for 60 min; and the caspase-3, VDAC1, and β-actin proteins at 200 mA for 80 min. The PVDF membrane was blocked in Tris-buffered saline (TBST) containing 5 % skim milk powder for 2 h at room temperature. The PVDF membrane was incubated with anti-cytochrome c (1:800 dilution), anti-bax (1:800 dilution), anti-bcl-2 (1:800 dilution), anti-caspase (1:300 dilution), anti-VDAC1 (1:200 dilution), and anti-β-actin antibodies (1:400 dilution) at 4 °C overnight. After incubation, the membrane was washed five times for 5 min each with TBST and then incubated with goat anti-rabbit IgG HRP conjugate (1:50000) for 2 h in TBST. The membrane was washed with TBST as described above and detected by ECL in accordance with the manufacturer’s protocol. The protein expression levels were analyzed by comparing the value of intensity_target_/intensity _endogenous control_.

### Total RNA extraction and RT-PCR of apoptosis-related genes

The total RNA of sperm samples was extracted by Trizol reagent in accordance with the manufacturer’s instructions. Specific primers for *bcl-2*, *bax*, *cytochrome c*, and *cas-3* genes were designed and synthesized by GenScript (Nanjing) Co., Ltd. (Nanjing, China) (Table 1). RT-PCR was conducted with the Vii™7 QRT-PCR (ABI, USA) and SYBR Green/Flourescein qPCR Master Mix (2X). RT-PCR reactions of *β-actin*, *bcl-2*, *bax*, *cytochrome c*, and *caspase-3* genes were conducted under the following conditions: one cycle of 50 °C for 2 min, 95 °C for 10 min, followed by 40 cycles of 95 °C for 30 s and 60 °C for 30 s. *β-actin* was used as the endogenous control. The relative amount of the target gene was calculated by the following formula: Fold change = 2^− ΔΔCt^, ΔΔCt = (Ct_target_ − Ct_β − actin_) − (Ct_target_ − Ct_β − actin_)_control_ [31, 32].

**Table 1:** The details of primer of β-actin and the target genes for RT-PCR

Gene	Primers(5′-3′)	Product(bp)
*β-actin*	F: CACGATGGAGGGGCCGGACTCATC	240
	R: TAAAGACCTCTATGCCAACACAGT	
*bcl-2*	F: GGTGAACTGGGGGAGGATTG	197
	R: GCATGCTGGGGCCATATAGT	
*bax*	F: GGCGATGAACTGGACAACAA	151
	R: CAAAGTAGAAAAGGGCAACC	
*cytochrome c*	F: AAAGGAGGCAAGCATAAGACTGG	104
	R: CCTTTGTTCTTGTTGGCATCTGT	
*Caspase3*	F: GGACCTGTGGACCTGAAAAA	159
	R: GCATGCCATATCATCGTCAG	

### Data analysis

To obtain statistically meaningful results, all experiments were conducted immediately after rats being sacrificed. Data were expressed as mean ± SEM. The difference between the exposure and control groups was estimated by *t*-test with SPSS 19 (SPSS Inc., Chicago, USA). Statistical significance was considered at *P* < 0.05.

## Results

### Sperm count and malformation

No significant difference in the epididymal weights was observed between the control and exposure groups after 50 d of exposure to RF-EMR at 900 MHz. However, the body weights were significantly higher in the exposure group than the control. The ratio between the epididymal and body was significantly lower in the exposure group than in the control group (Table 2). The sperm count in the exposure group was significantly lower than that in the control group (Table 3). As shown in Table 3, no statistical differences in head, tail, and total malformation percentages were found between the exposure and control groups. However, the total number of malformed sperm was higher in the exposure group than in the control group. The percentages of malformed head were also higher in the exposure group than in the control group.

**Table 2:** The body and epididymis weights of male mice in control group and exposure group (*n* = 24; Mean ± SEM)

Groups	Body weights (g)	Epididymis weights (g)	Epididymis weight/Body weight (%)
control	446.04 ± 6.11	1.19 ± 0.01	0.27 ± 0.01
Exposure	474.68 ± 8.25*	1.14 ± 0.02	0.24 ± 0.01*

*P < 0.05 compared with the control group by using *t*-test

**Table 3:** The sperm count and percentage of sperm malformation of the male mice in control and exposure group (*n* = 24; Mean ± SEM)

Groups	Sperm count (×10^8^)	Head malformation (%)	Tail malformation (%)	Total malformation (%)
control	1.21 ± 0.08	7.97 ± 0.22	12.25 ± 0.33	20.22 ± 0.49
Exposure	1.10 ± 0.08*	8.18 ± 0.34	12.63 ± 0.25	20.81 ± 0.52

*P < 0.05 compared with the control group by using *t*-test

### Morphologic study of rat sperm

**Fig. 2:**
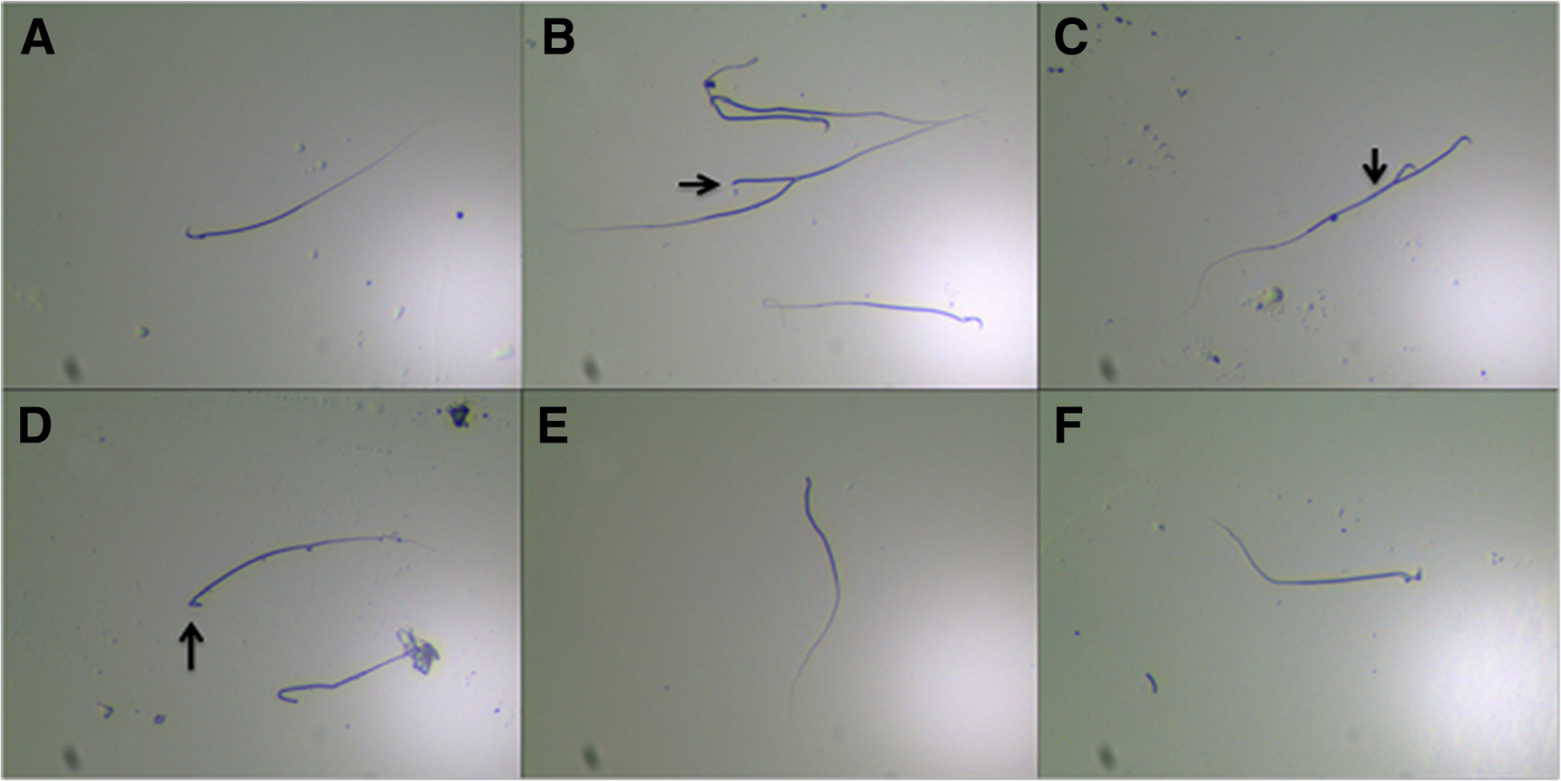
The optical micrographs of morphology of normal and malformed sperms. **a** The normal sperm. **b** The sperm cell with two tails. **c** The sperm cell with two heads. d The sperm cell with sharp head. **e** The sperm with no head. **f** The sperm cell with abnormal head

**Fig. 3:**
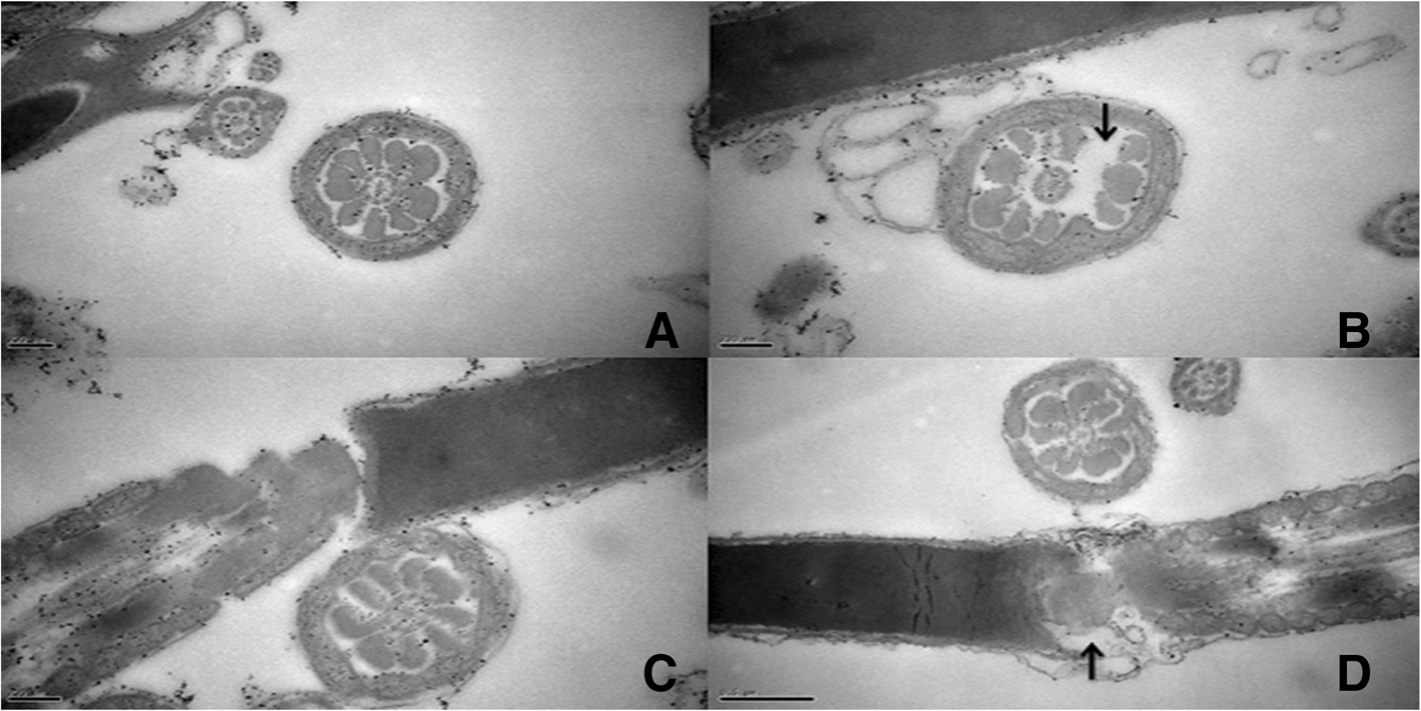
Electron micrographs of sperm necks and tails. **a** The cross section of sperm tail from control group. **b** The ODF was lost in the sperm tail of exposure group mouse as the arrow pointing. **c** The normal linked structure between the head and tail in the sperm of control group. **d** the linked structure of the sperm in exposure group was swollen as arrow pointing

Optical micrographs showed five types of sperm deformities in the heads and tails of the exposure group. In type 1, one sperm cell had two tails (Fig. 2). In type 2, one sperm cell had two heads (Fig. 2). Cuspidal sperm (type 3, Fig. 2), sperm with no head (type 4, Fig. 2), and sperm with abnormal head (type 5, Fig. 2) were also found in the exposure group. TEM was applied to observe the ultrastructural deformities in sperm (Fig. 3). The outer dense fibers (ODF) were absent (Fig. 3) in the sperm of the exposure group compared with the control (Fig. 3), and distension in the necks of sperm was observed in the exposure group (Fig. 3).

### Cell apoptosis and ROS and TAC levels in sperm

The apoptotic sperm cells were determined by flow cytometry (Table 4). The percentage of apoptotic sperm was significantly higher in the exposure group (22.47 % ± 1.83 %) than in the control group (11.51 % ± 0.53 %). The ROS level of sperm was significantly higher in the exposure group (53.00 ± 1.89/mg protein) than in the control group (36.51 ± 3.64/mg protein). However, the TAC level of sperm was significantly lower in the exposure group (4.80 ± 0.12 IU/mg protein) than in the control group (6.82 ± 0.21 IU/mg protein).

**Table 4:** The percentage of sperm cell apoptosis, the Value of the TAC and ROS in sperm suspension (*n* = 24, Mean ± SEM)

Groups	Apoptosis (%)	TAC (IU/mgpro)	ROS (OD/mgpro)
Control	11.51 ± 0.53	6.82 ± 0.21	36.51 ± 3.64
Exposure	22.47 ± 1.83*	4.80 ± 0.12*	53.00 ± 1.89*

*P < 0.05 compared with the control group by using *t*-test

### Western blot of cytochrome c, bal-2, bax, and active caspase-3

**Fig. 4:**
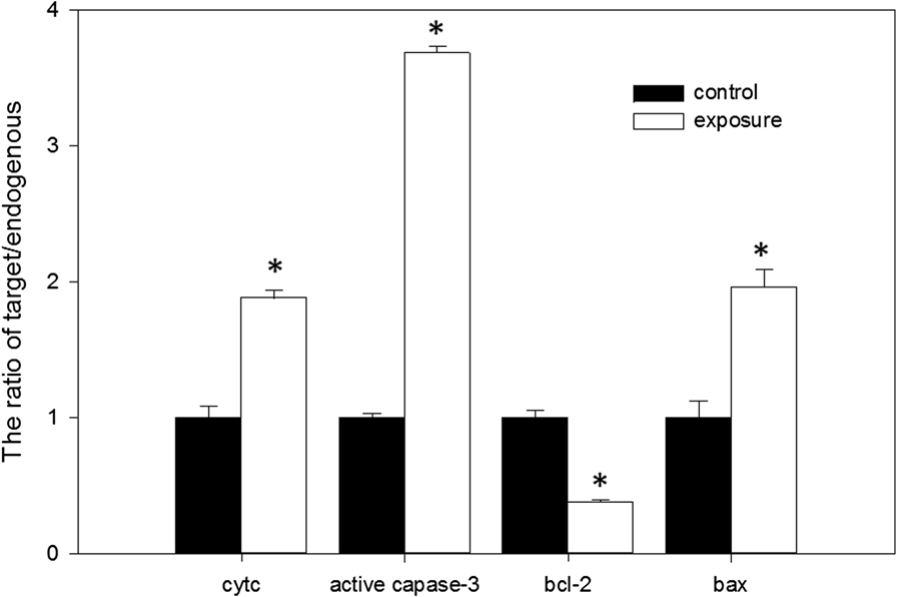
The comparison in expressions of cytochrome c, bcl-2, bax and caspase-3 between the exposure and control. Densitometric analyses of cytochrome c, bcl-2, bax and caspase-3 in sperm of rats between exposure group and control group. The intensities of bcl-2, bax and caspase-3 were normalized with the β-actin and the intensity of cytochrome c was normalized with VDCA1. Bars represent Mean ± SEM (*n* = 15). *indicated a significant difference at *P* = 0.05

**Fig. 5:**
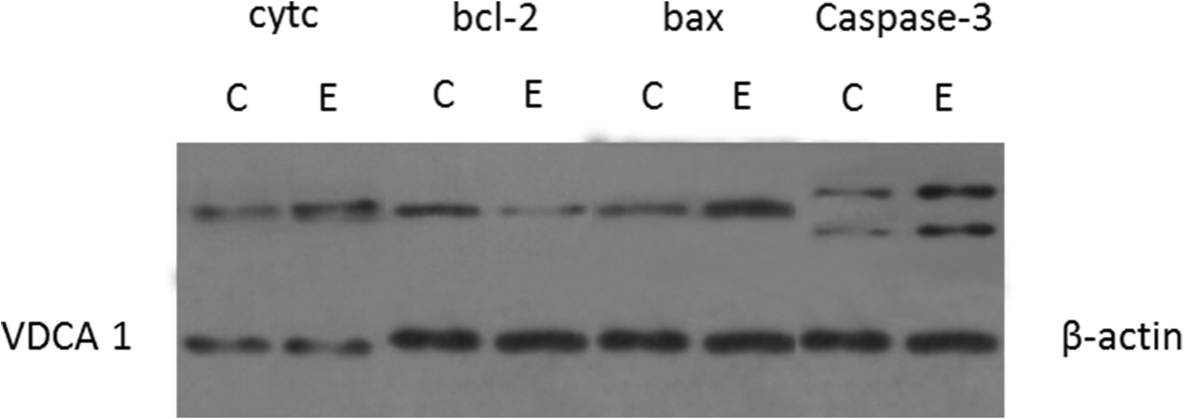
The electrophoresis results of cytochrome c, bcl-2, bax and caspase-3. C represented control group and E represented exposure group. There were two bands in the gel of caspase-3 indicating the inactive (upper) and active (lower) caspase-3 proteins. The gel below the gel of cytochrome c protein represented VDCA1 and other 3 gels represented the β-actin

To characterize the mechanism of RF-EMR-induced sperm apoptosis, the protein levels of cytochrome c, bcl-2, bax, and active caspase-3 were analyzed by western blot to characterize the mechanism of RF-EMR-induced sperm apoptosis. Compared with the sperm cells in the control group, those in the exposure group had significantly lower *bcl-2* expression (0.38 ± 0.01) while significantly higher *bax* (1.96 ± 0.13), *cytochrome c* (1.88 ± 0.05), and *active caspase-3* (3.68 ± 0.05) expression (Fig. 4). The electrophoresis results of cytochrome c, bcl-2, bax and caspase-3 were represented as Fig. 5. In Fig. 5, there were two bands in the results of caspase-3. The upper band is inactive caspase-3 and the lower band is the active caspase-3.

### Quantification of *bcl-2*, *bax*, *cytochrome c*, and *caspase-3* gene expression

**Fig. 6:**
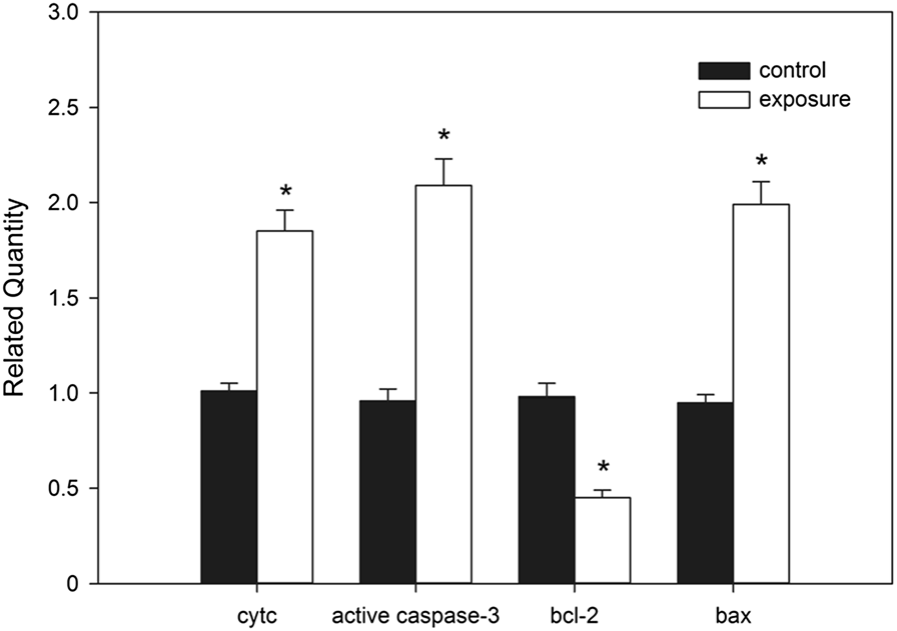
The related expressions of *bcl-2, bax, cytochrome c* and *caspase-3* genes in the sperms of the rats with different groups. The data was presented as Mean ± SEM (*n* = 15). *presented significant difference compared with the control group (*P* < 0.05)

The relative expression levels of the *bcl-2*, *bax*, *cytochrome c*, and *caspase-3* genes in the sperm of the rats from the control and exposure groups are presented in Fig. 6. The expression of the *bcl-2* gene was significantly lower in the exposure group (0.45 ± 0.04) than in the control (0.98 ± 0.07). The expression levels of *bax*, *cytochrome c*, and *caspase-3* were significantly higher in the exposure group (1.98 ± 0.12, 1.85 ± 0.11, and 2.09 ± 0.14 respectively) than in the control group (0.95 ± 0.04, 1.01 ± 0.04, and 0.96 ± 0.06, respectively).

## Discussion

Previous studies demonstrated that there were no difference in epididymal weight between rats exposed to RF-EMR and control[33]. The present study found similar results. However, contradictory results were obtained with regard to the effect of RF-EMR on sperm count. Dasdag et al. found that rats exposed to RF-EMR emitted by mobile phones and control rats showed no considerable difference in sperm count [21]. Vignera et al. found that Leydig cells, seminiferous tubules, and spermatozoa were the main targets of damage caused by mobile phones on the male reproductive tract. In particular, cellular phone exposure impaired spermatogenesis and damaged sperm DNA [28]. In the present study, sperm count was significantly lower in the exposure group than in the control group. This result agrees with a previous report [20].

In the present study, the percentage of abnormal sperm cells did not significantly differ between the exposure and control groups. However, the percentage of malformed sperm cells was higher in the exposure group than in the control group. Moreover, sperm cells with two heads or two tails were detected in the exposure group. Many studies revealed that RF-EMR does not significantly affect rat sperm morphology. However, only a few studies have investigated the effect of RF-EMR on sperm ultrastructure. The present study discovered that continuous RF-EMF changed the sperm ultrastructure in rats. The absence of ODF and distension in the neck of the sperm cells exposed to 900 MHz EMR corroborated the toxic mechanism of EMR-induced oxidative stress.

Oxidative stress may be a potential route for reproductive dysfunction [34]. Sperm function and male infertility are related to ROS-induced oxidative stress [35–37]. ROS can directly attack unsaturated fatty acids on the sperm membrane, induce lipid peroxidation, damage membrane integrity, destroy axoneme structure, and eventually reduce sperm activity and fertility [35, 37, 38]. Enzymatic antioxidants such as SOD, catalase, and GPX protect sperm from oxidative attack [39]. Previous investigation revealed that RF-EMR decreases GPX and SOD levels, and significantly increases catalase activity in rats [23]. The alteration in enzymatic antioxidant level may elevate ROS, which can activate the cell apoptotic signaling cascade. Excessive apoptosis can result in male infertility. In the present study, the percentage of apoptotic cells in the exposure group significantly increased by 91.42 %. The ROS concentration increased by 46.21 %. The TAC value, which is the sum of enzymatic and nonenzymatic antioxidants, decreased by 28.01 %, which may result in the male infertility associated with RF-EMR. In conclusion, increased apoptosis, elevated ROS, and decreased TAC contribute to male infertility [38].

The bcl-2 family proteins (bcl-2, bax), caspase family proteins (caspase-3), and cytochrome c are important regulatory proteins in cell apoptosis [40]. Cell apoptosis is inhibited by bcl-2, which can prevent bax gene expression and inhibit free radical generation. Bax is an important protein that determines cell apoptosis. The pro-apoptotic mechanism of *bax* is performed by promoting the release of cytochrome c, which can activate caspase-3 and form a dipolymer with bcl-2. The release of cytochrome c after mitochondrion damage results in DNA damage and apoptosis by activating the caspase-mediated pathway [38, 40]. Therefore, the relationship between the apoptosis caused by RF-EMR and the changes in bcl-2, bax, cytochrome c, and active caspase-3 expression is intriguing. In the present study, the protein expression levels of bax, cytochrome c, and active caspase-3 significantly increased while the expression level of bcl-2 decreased in the sperm of the rats in the exposure group. This result indicates that RF-EMR induced sperm apoptosis through bcl-2, bax, cytochrome c and active caspase-3 signaling pathways. The decreased expression levels of bcl-2 explained the increased levels of ROS.

In the present study, the different mRNA expression levels of the *bcl-2*, *bax*, *cytochrome c*, and *caspase-3* genes confirmed the western blot results. Together the RT-PCR and western blot results indicated that 900 MHz RF-EMR may elevate the concentration of ROS in sperm and consequently alter the expression of the genes related to cell apoptosis. The alteration in the expression of the genes related to sperm apoptosis caused the reduction of sperm count.

MRI and NMR are non-invasive technique widely used for clinical diagnosis and structural analysis [41]. There are three different electromagnetic fields emitted by MRI or NMR: static magnetic field, time-varying gradient magnetic fields and pulsed RF fields [42, 43]. Many articles indicated that there was no DNA damage from electromagnetic radiation of NMR or MRI. However, only limited investigations have examined whether *in vitro* and/or *in vivo* exposure of cells to electromagnetic fields used in MRI can cause changes of micronuclei [44], single strand breaks [45] and double strand breaks [46]. In this article, the frequency of RF was similar to the NMR. Hence, the investigations about expression of apoptosis-related genes caused by RF emitted by NMR or MRI would be an intriguing future study.

## Conclusions

This study demonstrates that RF-EMR can increase ROS levels and decrease TAC in the rat sperm. Excessive oxidative stress alters the expression levels of apoptosis-related genes and triggers sperm apoptosis through endogenous damage, resulting in male infertility.

## Abbreviations

ROS: Reactive oxygen species
TAC: Total antioxidant capacity
RT-PCR: Reverse transcription-polymerase chain reaction
EMR: Electromagnetic radiation
RF: Radiofrequency
HTF: Human Tubal Fluid
HRP: Horseradish peroxidase
ECL: Enhanced chemiluminescence
TEM: Transmission electron microscopy
PBS: Phosphate-buffered saline
DCFH-DA: Dichloro-dihydro-fluorescein diacetate
PVDF: Polyvinylidene fluoride
TBST: Tris-buffered saline
ODF: Outer dense fibers
SOD: Superoxide dismutase
GPX: Glutathione peroxidase
cytc: Cytochrome c
NMR: Nuclear magnetic resonance
MRI: Magnetic resonance imaging
